# Suppression of deflagration flame propagation of methane-air in tube by argon gas and explosion-eliminating chamber

**DOI:** 10.1038/s41598-022-09086-z

**Published:** 2022-03-23

**Authors:** Quan Wang, Xiaomeng Xu, Weida Chang, Zhimin Li, Jun Zhang, Rui Li

**Affiliations:** 1 School of Chemical Engineering, Anhui University of Science & Technology, Huainan, 232001 China; 2 Engineering Laboratory of Explosive Materials and Technology of Anhui Province, Huainan, 232001 China; 3 School of Civil Engineering and Architecture, Anhui University of Science & Technology, Huainan, 232001 China; 4 Fire & Explosion Protection Laboratory, Northeastern University, Shenyang, 110819 China

**Keywords:** Energy science and technology, Engineering

## Abstract

To explore the inhibitory effect of argon gas and explosion-eliminating chamber on methane-air deflagration flame propagation in the tube, based on the Φ = 120 mm, L = 5.5 m stainless steel pipeline test system to measure methane-air deflagration flame structure, flame propagation speed, and deflagration pressure. The results show that: 10–30% argon is mixed into the methane-air premixed gas with different equivalent ratios. With the increase in the mixed argon content, the tensile distortion and instability of the flame front increase, and the average value of flame propagation speed decreases by 2.52–60.0%. The first and second deflagration pressure peaks are reduced by about 13.1–62% and 17.7–86.5% respectively. The average value of the methane-air deflagration flame propagation velocity was reduced by 5.7–37.0% with the explosion-eliminating chamber laid at the nozzle. The second and third deflagration pressure peaks are reduced by about 10–30% and 50–90% respectively. The inhibitory effect of argon on the propagation of methane-air flame is considered better than the laying of the explosion-eliminating chamber under the experimental conditions.

## Introduction

Methane is widely used in daily production and life, but as a combustible gas, if it is not used properly, it may explode and cause serious harm. At present, the most commonly used method to prevent explosion hazards is to add certain inert medium in methane gas as flame retardant, such as nitrogen (N_2_), carbon dioxide (CO_2_) and water vapor (H_2_O)^1–3^. Inert medium can relatively reduce the volume fraction of combustible, so that it can be controlled beyond the explosion limit to achieve flame retardant effect. Kondo^4,5^ explored the effect of CO_2_ and N_2_ on the explosion limits of various combustible gases, and found that this effect can be explained by the extended Le Chatelier formula using a set of commonly used parameter values. Bundy^6^ studied the inhibition degree of inert gas CO_2_, N_2_ and trifluoromethane on explosion flame, and obtained the critical volume fraction of inert gas inhibiting flame. Benedetto^7^ studied the influence of CO_2_ content on the peak pressure, maximum pressure rise rate and laminar combustion rate, indicating that the main effect of CO_2_ is the effect on the specific heat of the mixture. When the CO_2_ content reduces the adiabatic flame temperature to about 1500 K, the flame will be extinguished. It is shown that the main effect of CO_2_ is on the specific heat of the mixture. When the CO_2_ content decreases the adiabatic flame temperature down to about 1500 K, the flame extinction occurs. Mashuga^8^ estimated the explosion limit of the mixture of methane/ethylene and nitrogen at the adiabatic flame temperature of 1200 K, and there was a good agreement between the results and the experimental values. Shebeko^9^ estimated the explosion limit of the ternary mixture of combustible and flame retardant at the adiabatic flame temperature of 1600 K, and proposed a new method for calculating the explosion limit of the mixture. Vidal^10^ calculated the adiabatic flame temperature by using SuperChems software to estimate the lower explosion limit of hydrocarbon/flame retardant mixture.

Besides, explosion venting as an effective disaster mitigation method has also been widely studied by scholars. Cooper^11^ explored the influence of vent area on the deflagration flame pressure peak. The first, third and fourth pressure peaks increase with the decrease of vent area, while the second pressure peak first increases and then decreases. Bauwens^12^ also found that the smaller vent area would lead to higher internal overpressure peak of the direct vented vessel. However, Ponizy^13^ also show that the increase of vent area does not always lead to the decrease of overpressure peak. Ferrara^14^ explained this phenomenon as the result of the interaction between combustion rate and venting rate. Solberg^15^ found that the pressure of central ignition is the highest and the pressure rising speed is the fastest in the pipe with vent. Bradley^16^ thought that the most dangerous ignition position in the spherical vessel with vent is in the center of the vessel. Kasmani^17^ studied the explosion overpressure of methane air premixed gas in the cylindrical vessel with vent, and the tail ignition can cause more serious consequences. Alexiou^18^ found that when the pressure relief port is close to the ignition end, the explosion relief effect is the best, and when the pressure relief port is located in the middle of the pipeline, the explosion relief effect is the worst.

However, in large pipelines, argon is used as a diluent gas to suppress the propagation of methane-air deflagration flame and the related research on the explosion suppression device is relatively rare. Based on this, this article explores and compares the inhibitory effect of argon gas and explosion-eliminating chamber on methane-air deflagration flame propagation in the tube, according to the flame motion images, flame propagation speed, deflagration pressure, and other characteristic parameters.

## Experimental section

### Materials

Methane (99.99%) and argon (99.999%) were purchased from Hefei Henglong Electric Co., Ltd, (Hefei, P. R. China).

### Experimental system

**Figure 1:**
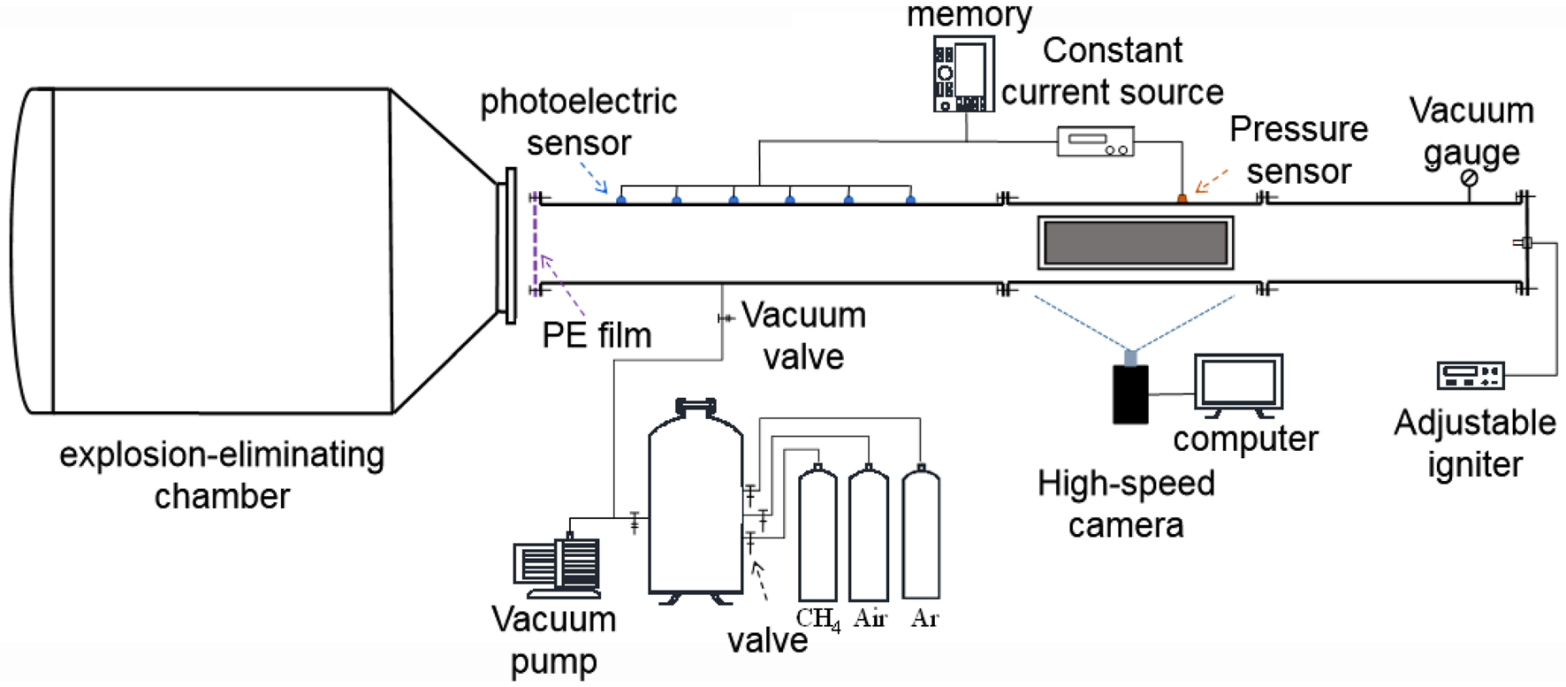
Experimental system.

As shown in Fig. 1, the experimental system consists of a stainless steel flame acceleration pipeline, a gas distribution system, a detonation chamber, a high-speed camera, and a data acquisition system. The flame acceleration tube is a cylindrical tube of Φ = 120 mm and L = 5.5 m, and an observation window of 40 cm $\times$ 7 cm is laid at 130 cm from the ignition end of the tube. The ignition end of the pipeline is closed, and the open end is closed with 2 layers of polyethylene (PE) film with a thickness of 0.02 mm.

A PCB pressure sensor (with a response time of fewer than 1 μs) is laid at 141 cm from the ignition end of the pipeline to monitor the deflagration pressure in the pipeline, while 6 flame sensors are laid at the pipeline with a horizontal distance from 226 to 476 cm (50 cm interval) from the ignition end to monitor the deflagration flame propagation velocity. The high-speed camera is used to monitor images of deflagration flames directly at the observation window at a shooting rate of 2000 fps and save them in a computer.

**Figure 2:**
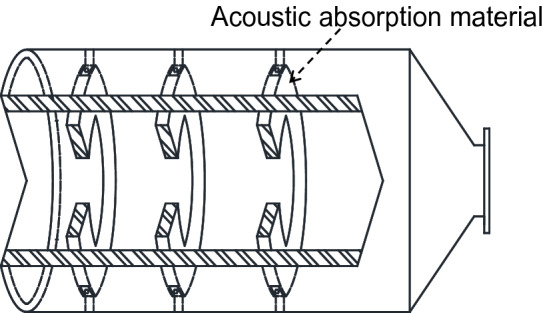
Structure diagram of explosion-eliminating chamber.

As shown in Fig. 2, the explosion-eliminating chamber used in the experiment is an explosion suppression device with a volume of 1.53 m^3^, and three explosion suppression rings are installed at equal distances inside the tank. The explosion suppression ring described in this article is polyester fiber cotton mainly consisting of annular porous acoustic absorbent material.

### Gas preparing scheme

The pipeline is first evacuated to vacuum and then filled with the configured methane-air premixed gas to make the pipeline pressure return to 1 atm for the experiment. The formula for calculating the equivalent ratio of combustible gas is: In the formula: *Ф* is the combustible gas equivalent ratio, and *m* is the gas mass. The gas composition required for the experiment is shown in Table 1. To ensure that the prepared gas is evenly mixed, it needs to be premixed in the air mixing tank for 3–5 h.

**Table 1:** Experimental gas preparing scheme for the suppression of methane-air deflagration flame in the explosion-eliminating chamber.

*Φ*	CH_4_ (vol.%)	Air (vol.%)
0.9	8.64	91.36
1.0	9.50	90.5
1.1	10.36	89.64

When exploring the inhibitory effect of argon added on the deflagration flame of methane-air in the tube, there is no reaction between argon and the methane-air mixture, so the argon ratio can be calculated from:

1 $$\beta = \frac{{V_{dilution} }}{{{\text{V}}_{{{\text{dilution}}}} + {\text{V}}_{{{\text{CH}}_{4} }} + {\text{V}}_{{{\text{air}}}} }} \times 100\%$$

Here, *V*_*dilution*_ is the volume of inert gas, *V*_*air*_ is the volume of air, and *V*_*CH4*_ is the volume of methane. The gas preparing scheme calculated is shown in Table 2 according to the above formula.

**Table 2:** The experimental gas distribution scheme of argon on the suppression of methane-air deflagration flame.

$\beta_{Ar} /\%$	*Φ* = 0.9			*Φ* = 1.0			*Φ* = 1.1		
	Ar/%	CH_4_/%	Air/%	Ar /%	CH_4_/%	Air/%	Ar/%	CH_4_/%	Air/%
0	0.00	8.64	91.36	0.00	9.50	90.50	0.00	10.36	89.64
10	10.00	7.78	82.22	10.00	8.55	81.45	10.00	9.31	80.69
20	20.00	6.91	73.09	20.00	7.60	72.40	20.00	8.29	71.71
30	30.00	6.05	63.95	30.00	6.65	63.35	30.00	7.25	62.75

## Experimental results and discussion

### Effect of argon on the flame propagation characteristics of methane-air deflagration

#### Effect of argon on deflagration flame profile

**Figure 3:**
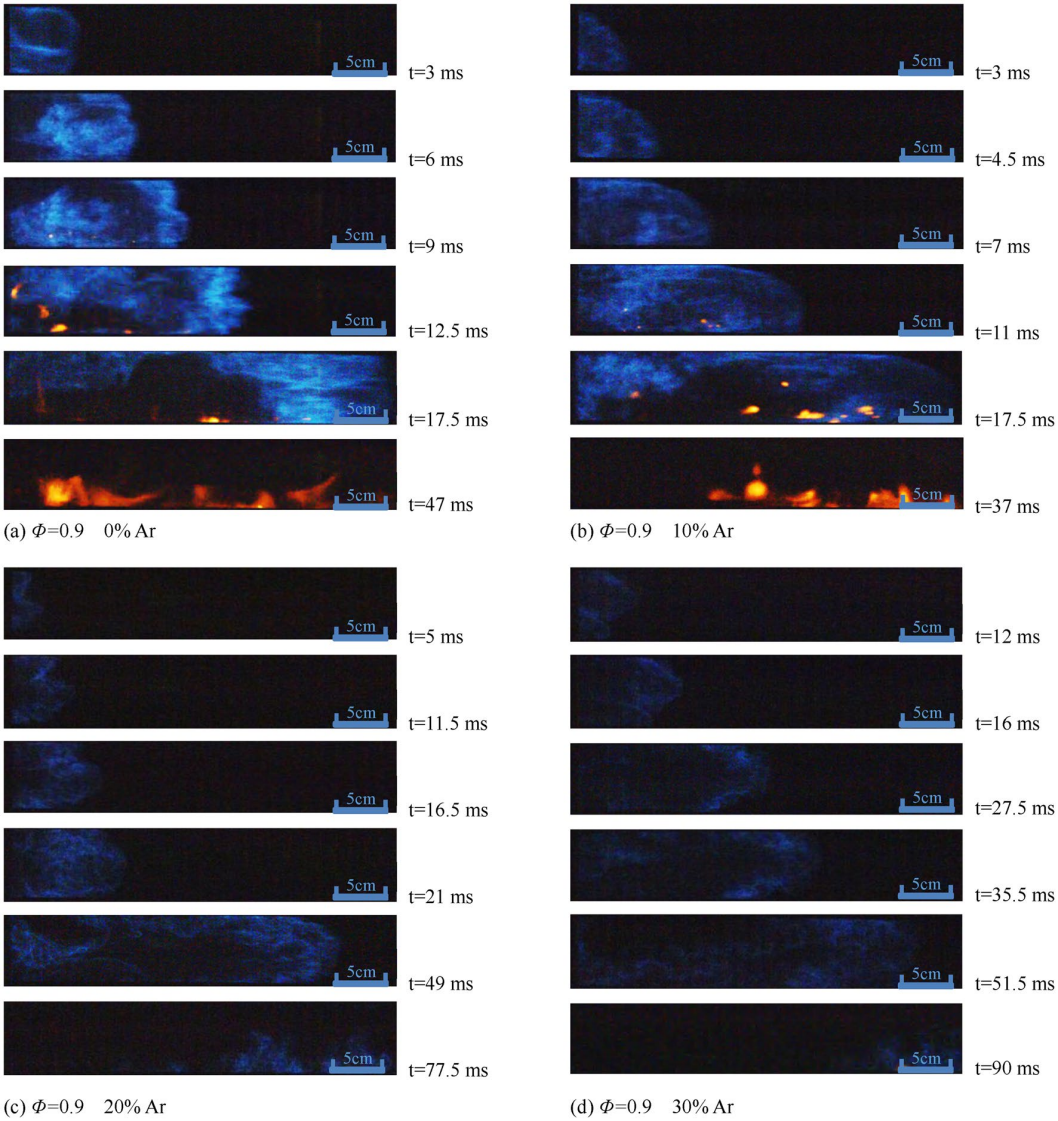
Flame structure with different proportion of Ar under the condition of *Φ* = 0.9.

**Figure 4:**
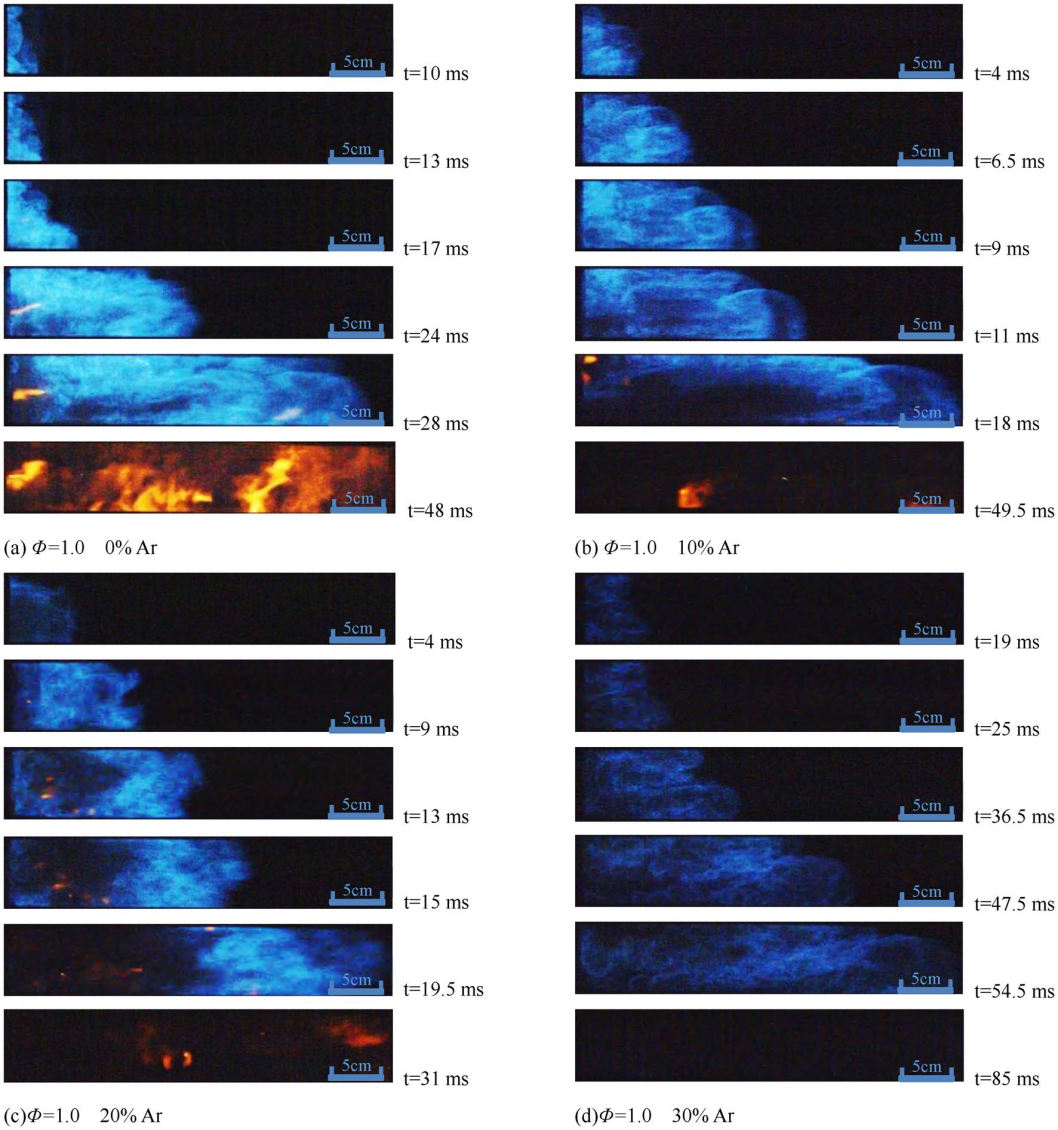
Flame structure with different proportion of Ar under the condition of *Φ* = 1.0.

**Figure 5:**
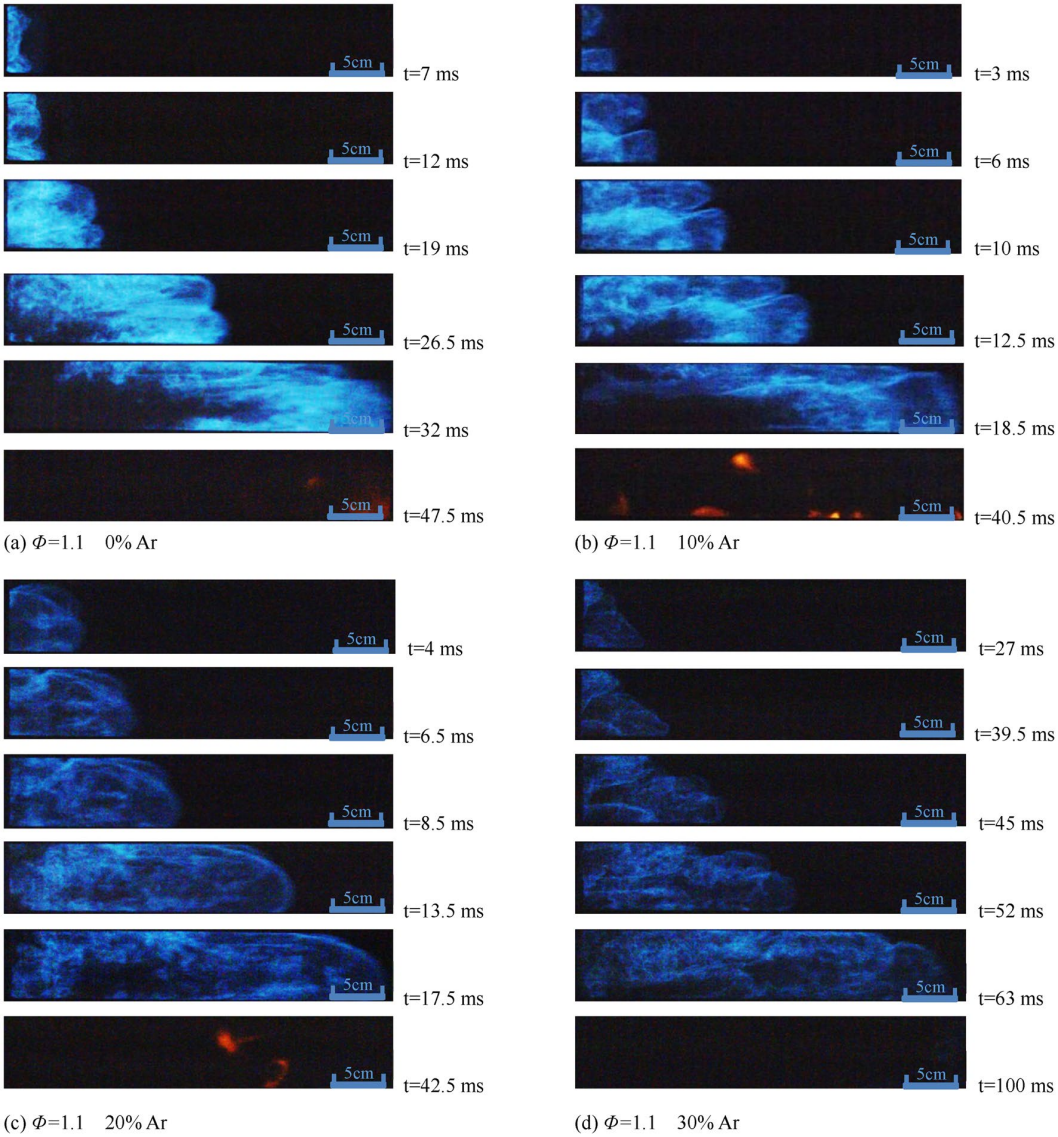
Flame structure with different proportion of Ar under the condition of *Φ* = 1.1.

Figures 3, 4 and 5 are photos of the typical deflagration flame profile captured in the experiment to explore the propagation, growth, and structural changes of the premixed flame under different experimental conditions. Assume that the previous picture before the flame front enters the observation window is t = 0 ms.

It can be seen from Fig. 3 that when argon content is 0, the front of the deflagration flame gradually changes from a "finger" to a flat surface, and then turns inward until it passes through the observation window because of the rise of pressure at t = 9 ms. With the increase of argon content, the flame front becomes irregular, the propagation time of the "finger-shaped" flame front increases, and the brightness of the premixed flame and the bright yellow flame in the later stage of the combustion gradually decrease.

It can be seen from Fig. 4 that the flame front is mostly a "protruding" structure. With the increase of argon content, the instability of the combustion increase, and the flame brightness decreases, while the brightness of premixed flame under *Φ* = 1.0 condition is higher than that under *Φ* = 0.9 condition under the same ratio of argon added.

As shown in Fig. 5, the flame front has been stretched to a certain extent as soon as it enters the observation window when argon content is 0. The internal concave phenomenon appears with the flame spreads, and then develops into a Tulip flame at t = 26.5 ms (Some photos are processed by image enhancement to highlight the Tulip flame characteristics).

Comparing the flame profile photos in Figs. 3, 4, and 5, the influence of argon added on the flame structure of methane-air premixed gas at *Φ* = 0.9, *Φ* = 1.0, and *Φ* = 1.1 decreases in order, which means that the lower the equivalent proportion is, the more obvious the dilution effect of the argon is, and the slower the combustion reactions are. Under the circumstance of same Φ, the flame brightness gradually decreases as the proportion of argon increases.

#### Effect of argon on the propagation velocity of methane-air premixed flame

**Figure 6:**
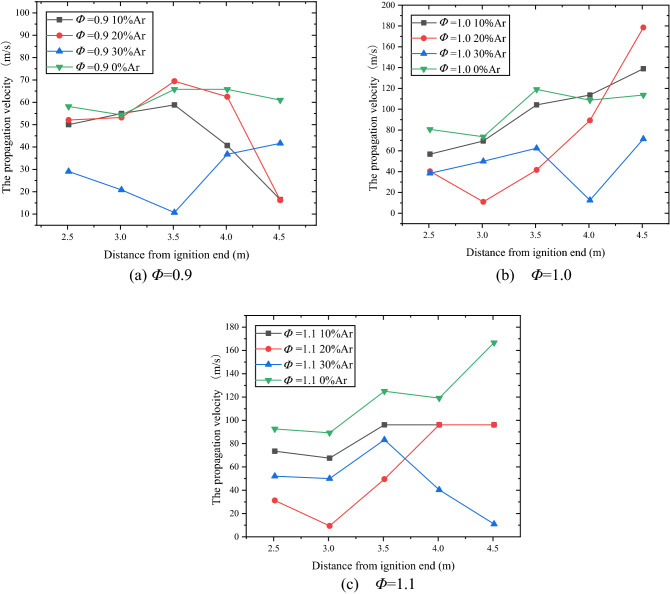
Flame propagation velocity with different ratio of Ar.

Figures 6 is graphs of flame propagation velocity in the "near open-end" section of the pipeline with different ratio of Ar. Taking the experimental condition of *Φ* = 0.9 (Fig. 6a) as an example, when argon is not added, the flame propagation velocity curve is relatively smooth, while the velocity curve becomes no longer smooth with the increase of the argon content. The development trend of the flame propagation velocity curve under the circumstance of *Φ* = 0.9 10% Ar is similar to that of *Φ* = 0.9 20% Ar, which shows a slow rise first and then a large decrease. But the curve under the circumstance of *Φ* = 0.9 30% Ar is completely different from the curves mentioned above, showing a development trend of rising first and then falling, and the average flame propagation velocity is the smallest under this experimental condition. The specific values are shown in Table 3.

**Table 3:** Research rule of argon suppression on methane-air deflagration flame propagation velocity curve under various experimental conditions.

$\beta_{Ar} /\%$	Average speed (m/s)			Speed decreasing rate (%)		
	Φ = 0.9	Φ = 1.0	Φ = 1.1	Φ = 0.9	Φ = 1.0	Φ = 1.1
0	61.0	99.1	118.5	–	–	–
10	44.2	96.6	85.9	27.5	2.52	27.5
20	50.7	72.2	56.5	16.8	27.2	52.3
30	27.8	46.9	47.3	54.4	52.6	60.0

It can be seen from Fig. 6b that under the experimental conditions of *Φ* = 1.0 0% Ar and *Φ* = 1.0 10% Ar, the development trend of the velocity curves are similar, and the average flame propagation velocity under 10% Ar condition is slightly lower than that under no argon condition. With the further increase of the argon content, the trend of the speed curve begins to change under the *Φ* = 1.0 20% Ar experimental condition, and the velocity curve of *Φ* = 1.0 30% Ar condition is obviously lower than the other three curves, indicating that the argon content of 30% has a relatively obvious effect on reducing the flame propagation speed.

#### Effect of argon on flame deflagration pressure of methane-air deflagration

**Figure 7:**
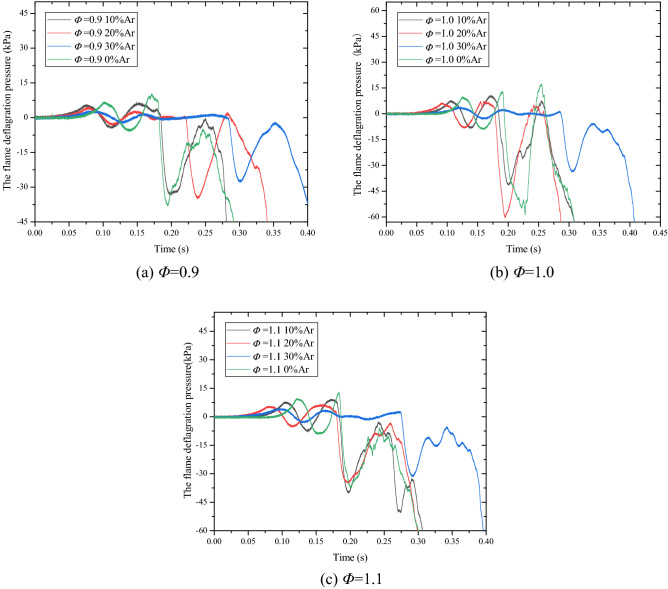
The time curves of the flame deflagration pressure with different ratio of Ar.

Figures 7 is time curves of the flame deflagration pressure in the pipeline with different ratio of Ar. It can be seen from the figures that the development trend of the other deflagration pressure–time curves is the same except for the three experimental conditions of *Φ* = 0.9 30% Ar, *Φ* = 1.0 30% Ar, and *Φ* = 1.1 30% Ar.

In the experiment, two layers of PE film are used as the constraining material at the open end of the pipeline under each experimental condition, and its rupture pressure and material strength, and other parameters are conformed to the following relations^19^:

2 $$\Delta P = \frac{\delta \sigma }{d}$$

Here, Δ*P* is the pressure difference between the two sides of the diaphragm, *δ* is the thickness of the diaphragm, σ is the tensile strength, *d* is diameter of the pressure relief port.

The PE film used in the experiment is 0.02 mm, and its factory-marked tensile strength is about 20 MPa. It can be estimated that the rupture pressure of the first layer is 3.3 kPa and the rupture pressure of the second layer is 6.6 kPa according to the above formula.

**Figure 8:**
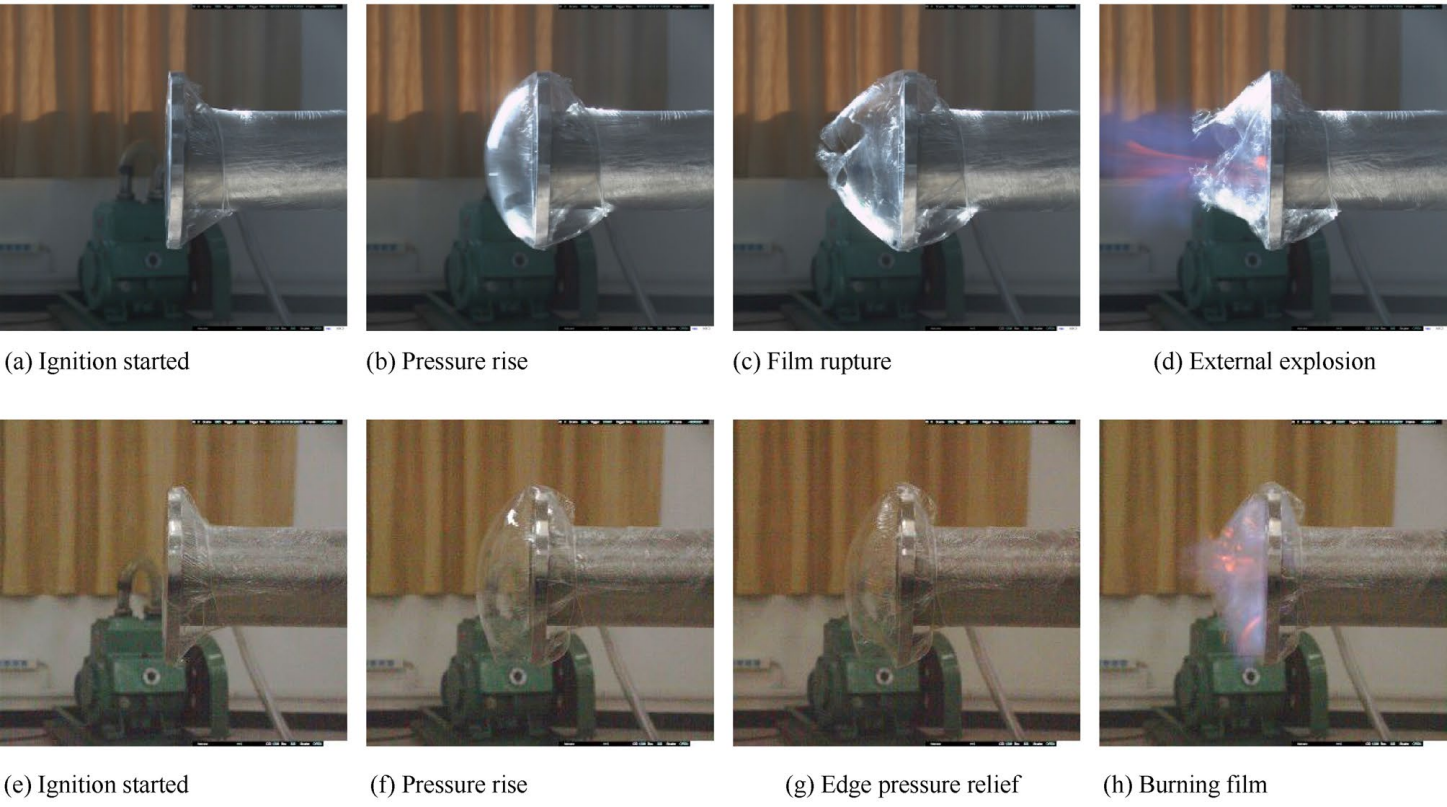
High speed camera of typical film breaking process.

High-speed images of a typical film breaking process are shown in Fig. 8. Taking the experimental condition of *Φ* = 0.9 (Fig. 7a) as an example, when the premixed gas in the tube is ignited without breaking the film (Fig. 8b), the pressure in the tube gradually rises and the first pressure peak appears, that is film rupture pressure.

Then the flame turbulence increases, and the deflagration pressure starts to rise due to the change in the flow field of the premixed gas in the tube caused by the film rupture. The flame front reaching the open end of the tube will ignite the unburned premature gas that rushed out of the pipeline after the film was broken. The mixed gas ignites to form an "external explosion" (Fig. 8d), which hinders the release of gas in the pipeline and generates a reverse pressure wave, prompting a rapid increase in deflagration pressure, resulting in a second pressure peak.

When the "external explosion" ends, the combusted gas is quickly released to the outside of the tube, causing negative pressure in the tube and triggering the oscillation of the combustible gas in the tube^20^. This oscillation will cause Taylor instability and further intensify the turbulence degree of the flame in the tube^11^, and finally leads to the appearance of the third pressure peak. The pressure in the tube will eventually return to normal pressure as the combustion reaction ends.

With the further increase of the argon content, the first pressure peak dropping significantly under the Φ = 0.9 20% Ar experimental condition is 4.70 kPa, which is not enough to break through the film. Then the flame pressure is relieved from the interface between the film and the tube opening, that is, "edge pressure relief" (Fig. 8g). With the further reaction of the unburned gas in the tube, the pressure rises slightly and the second peak pressure appears, and the final pressure curve remains steady until the flame propagates to the end and burns through the film. After the "burning film" shown in Fig. 8h, the pressure of the flame rushing out of the pipeline drops rapidly. When the content of argon reaches 30%, the first pressure peak value decreases to 2.53 kPa. At this time, the pressure is not enough to break through the film of the nozzle, and "edge pressure relief" occurs. The second pressure peak is 1.38 kPa until the flame propagates to the end and burns through the film. And the pressure of the "burning film" flame rushing out of the tube drops rapidly the same as shown in Fig. 8h. The specific values are shown in Table 4.

**Table 4:** Research rule of argon suppression on methane-air deflagration flame propagation time curve under various experimental conditions.

*Φ*	$\beta_{Ar} /\%$	The first pressure peak (kPa)	The second pressure peak (kPa)
0.9	0	6.58	10.22
	10	5.72	6.58
	20	4.70	2.43
	30	2.53	1.38
1.0	0	9.50	12.64
	10	7.45	10.40
	20	6.23	6.81
	30	3.61	2.07
1.1	0	9.35	12.47
	10	7.79	8.83
	20	5.65	5.91
	30	4.31	3.29

### Effect of explosion-eliminating chamber on the flame propagation characteristics of methane-air deflagration

#### Effect of explosion-eliminating chamber on the Profile of deflagration flame

**Figure 9:**
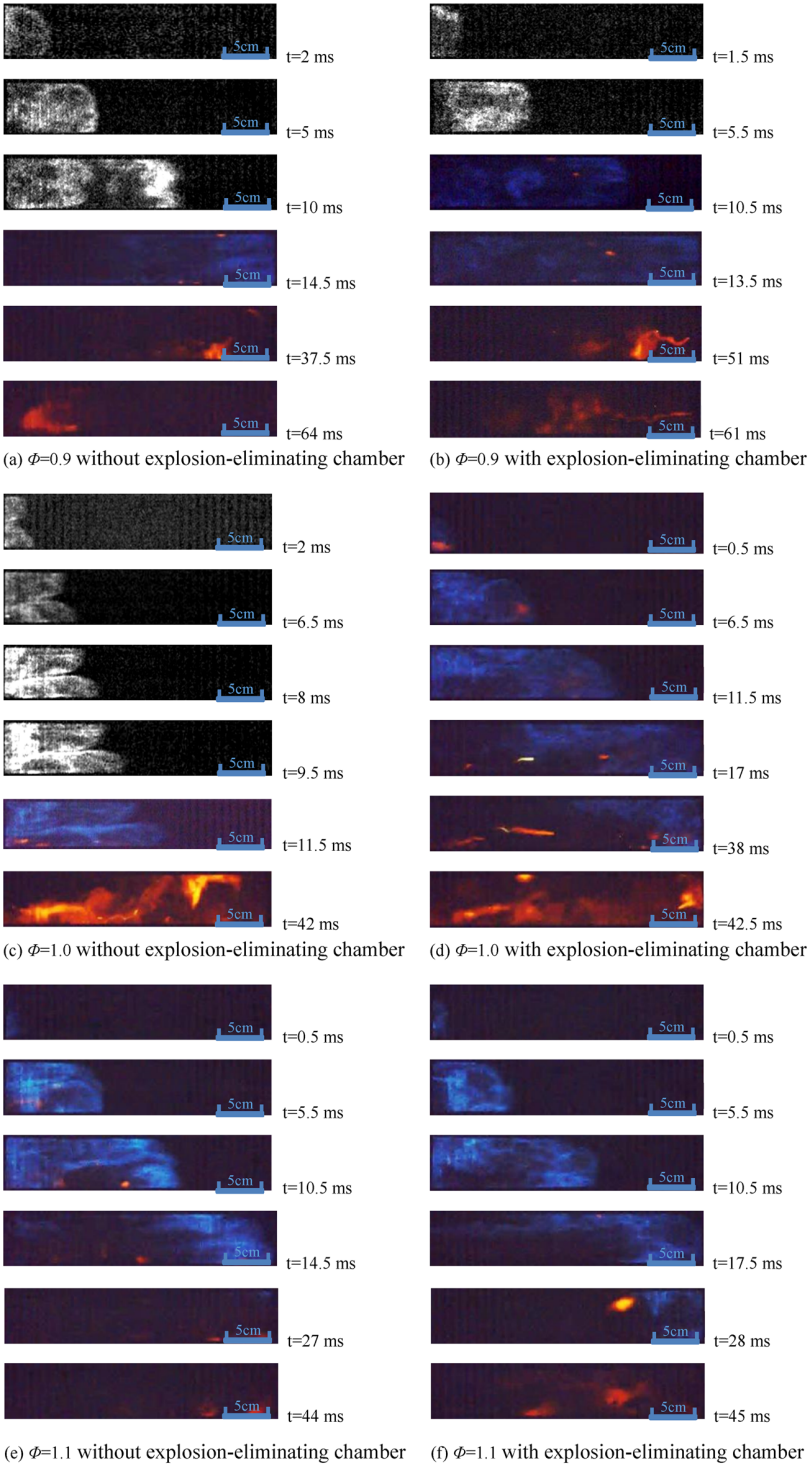
High-speed photographs of flame propagation under different experimental conditions.

The photos of the Profile of the deflagration flame at typical moments under each experimental condition are monitored in Fig. 9. It is assumed that the previous photo when the flame front enters the observation window corresponds to t = 0 ms (Monochrome photos are processed by image enhancement to highlight the Tulip flame characteristics).

As shown in Fig. 9a, when the flame front enters the observation window, the speed of flame propagation is relatively small at the initial stage, which is approximately laminar combustion. The flame front propagates forward as a spherical surface and then becomes a plane surface at t = 5 ms, where the flame superficial area is minimum. The flame shows a tensile deformation at t = 10 ms, and Tulip Flames are gradually formed. It can be seen from Fig. 9c that a typical Tulip Flame has been formed at t = 8 ms, and the flame wavefront is further stretched at t = 9.5 ms, significantly increasing the flame superficial area, intensifying the combustion reaction process, and inducing the transformation of laminar flame to turbulent flame. According to Markstein's model^21^, local propagation velocity of fold flame front can be calculated from the following equation:

3 $$v = v_{{\text{u}}} \left[ {1 + L\left( {\frac{1}{{R_{{{\text{flame}}}} }} - \frac{1}{{R_{{{\text{flow}}}} }}} \right)} \right]$$

where *v* and *v*_u_ are the local propagation velocity of the fold flame front and the laminar flame propagation, *L* is the characteristic length, *R*_flame_ and *R*_flow_ are the radius of curvature at the flame front and the curvature radius of flow field, respectively.

Combining Fig. 9c with the Markstein model, it can be seen that the flame spreads at a laminar velocity as a whole when *R*_flow_ and *R*_flame_ are the same. But when *R*_flow_ and *R*_flame_ are different, the local flame velocity will change, which will lead to changes in the flame surface structure. The interaction between the flow change of the flow field in the tube and the flame wavefront is an important reason for the formation of the Tulip Flame^22^.

The flame wavefront under other conditions in Fig. 9 has already been stretched and distorted in a large degree when entering the observation window, and the flame folds have become extremely irregular. The reason for the differentiation of the external characteristics during the flame propagation process might be different equivalence ratio conditions and the unevenness of the tube inwall and other factors^20,23^. The explosion-eliminating chamber mainly acts on the external flame explosion process after the film is broken, and will not have a significant impact on the initial flame structure.

#### Effect of the presence or absence of explosion-eliminating chamber on the propagation speed of deflagration flame

**Figure 10:**
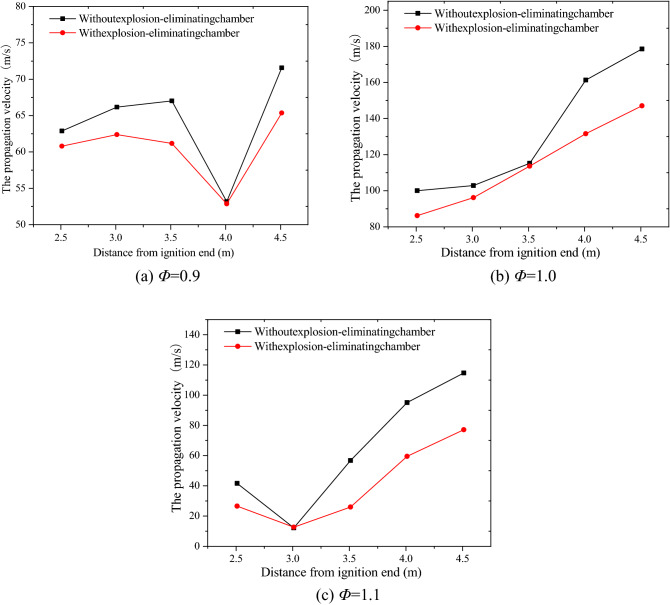
The effect on flame propagation velocity of laying explosion-eliminating chamber.

**Figure 11:**
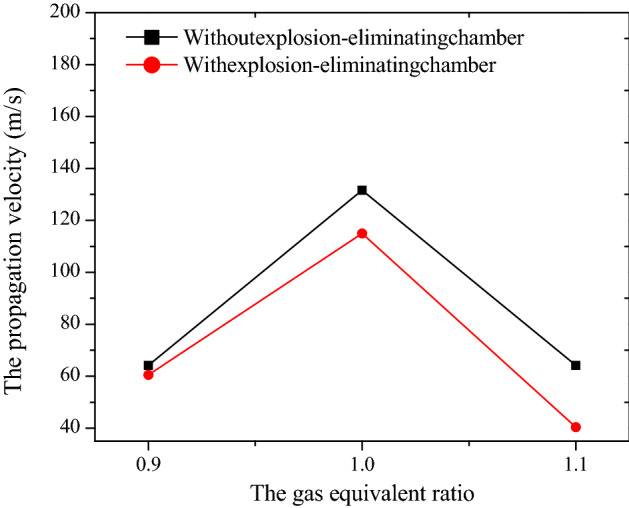
Flame propagation average velocity of different experimental conditions.

The effect on flame propagation velocity of laying explosion-eliminating chamber is shown in Fig. 10. It can be seen that the trend of the deflagration flame velocity curve under each experimental condition is gradually increasing. This is because more and more combustible gas are involved in the reactions, and flame turbulence has increased as the combustion reaction progresses, which causes the combustion reaction to be more violent. And the speed curves of the laid explosion-eliminating chamber were all located below the curve without the chamber, indicating that the laying of the chamber has a certain limiting effect on flame propagation. The curve of the propagation average velocity of the deflagration flame under all experimental conditions is shown in Fig. 11. The propagation velocity of the deflagration flame is the largest when *Φ* = 1.0. This is because the chemical reaction of the methane-air premixed gas under the theoretical equivalent ratio is the most complete and the combustion reaction is the most violent. The deflagration flame propagation speed under each experimental condition is reduced by 5.7%, 12.7%, and 37.0% respectively compared with the explosion-eliminating chamber conditions, indicating that the laying of the explosion-elimination chamber can significantly attenuate the flame propagation speed. Three explosion suppression rings (annular acoustic absorbing materials) laid in the chamber have a certain energy absorption and buffering effect on the gas release in the pipeline^24^, which will reduce the disturbance of the flow field and then the turbulence of flame propagation in the tube, and ultimately reduce the flame propagation speed in the pipeline^22^. The specific values are shown in Table 5.

**Table 5:** Research rule of argon suppression on methane-air deflagration flame propagation velocity curve under various experimental conditions.

*Φ*	Without explosion-eliminating chamber average speed (m/s)	With explosion-eliminating chamber average speed (m/s)	Speed decreasing rate (%)
0.9	64.14	60.51	5.7
1.0	131.59	114.92	12.7
1.1	64.15	40.39	37.0

#### Effect of explosion suppression chamber on deflagration pressure

**Figure 12:**
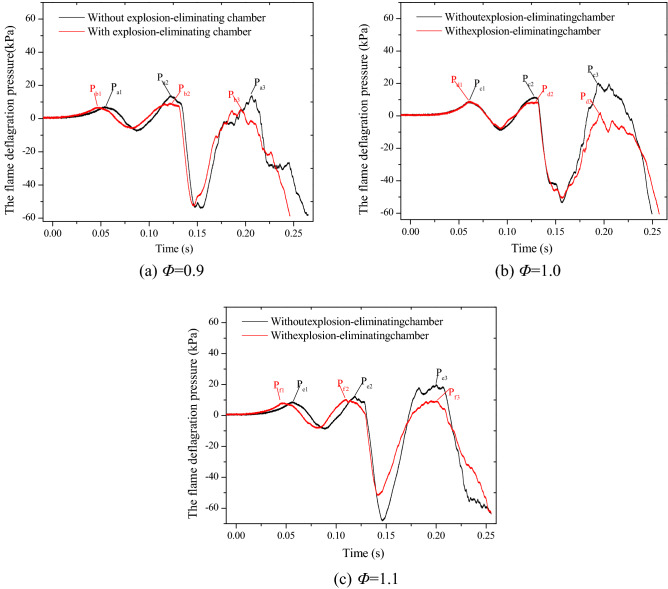
The effect on flame deflagration pressure of laying explosion-eliminating chamber.

The effect on flame deflagration pressure of laying explosion-eliminating chamber is shown in Fig. 12. Taking the experimental condition of *Φ* = 0.9 (Fig. 12a) as an example, when the explosion-eliminating chamber is laid, the overall trends of the deflagration pressure change and the physical process of reaching each peak pressure are basically similar to these without the chamber. However, there is a marked decline in the second peak pressure P_b2_ (9.3 kPa) and the third peak pressure P_b3_ (5.3 kPa) due to the laying of the chamber. On the one hand, the second pressure peak is reduced because of the deflagration flame at the tube mouth is quenched by the porous structure of the explosion suppression ring inside the explosion-eliminating chamber to a certain extent^25^. On the other hand, the deflagration flame pressure is significantly reduced by the annular acoustic absorbing material^24^. The pressure generated by the "external explosion" undergoes multiple reflections and attenuation among the three explosion suppression rings in the explosion-eliminating chamber. The third peak pressure is reduced because of the attenuation of the pressure wave causing the oscillation of the flow field in the pipeline, which weakens the turbulence degree of flame propagation in the pipeline. In general, the second and third pressure peaks will be respectively reduced by about 10% to 30% and 50% to 90% due to the laying of the explosion-eliminating chamber.

### Comparative analysis of the suppression effect of argon gas and explosion-elimination chamber on methane-air deflagration flame

From the point of view of the suppression effect of argon and the explosion-eliminating chamber on the flame propagation speed, it can be found that adding 10–30% argon in the methane-air premixed gas with different *Φ* reduces the flame propagation speed by 2.52–60.0%, an average reduction of 24.7%, while the laying of the explosion-eliminating chamber at the open end reduces the propagation speed of methane-air deflagration flames with different *Φ* by 5.7–37.0%, an average reduction of 18.4%. Thus it can be seen that the inhibitory effect of argon on the flame velocity is better than that of the explosion-eliminating chamber under the experimental conditions.

From the point of view of the suppression effect of argon gas and the explosion-eliminating chamber on the detonation pressure, it can also be found that the suppression effect of argon gas is better than that of laying the explosion-eliminating chamber. The suppression effect of argon on the detonation pressure exists from the beginning of ignition, and with the increase in argon content, the "Rupture pressure" is significantly reduced and "burning film" occurs. It can be seen from the pressure curve that the second and third pressure peaks after the film ruptures are suppressed by laying the explosion-eliminating chamber.

The analysis of the experiment shows that the suppression effect of argon on the methane-air flame propagation is better than that of laying an explosion-eliminating chamber. Nevertheless, the time when combustible gas will explode is hard to be predicted in actual production activities, so the inert gas such as argon cannot be injected in time to suppress combustion and explosion. However, explosion-eliminating chamber can be laid in advance at the explosion vents of production equipment, especially when the vents cannot be directly led to the outdoors. From this point of view, explosion-eliminating chamber might be more suitable for practical production than inert gases.

## Conclusion

In the study, the inhibitory effect of argon gases and explosion-eliminating chamber on methane-air deflagration flame were explored by using the flame acceleration pipeline test system. It can be concluded that the inhibitory effect of argon on the methane-air flame propagation is better than that of laying the explosion-eliminating chamber under the experimental conditions, and the following main conclusions were obtained.

1. The flame propagation can be significantly inhibited by adding argon in methane-air premixed gas, and when the equivalent ratio *Φ* is constant, the tensile distortion and instability of the flame front increase with the increase of the mixed argon content, and the brightness of the premixed flame decreases as well.
2. The average speed of flame propagation and the deflagration pressure can be significantly reduced by adding argon in methane-air premixed gas, especially when the content of argon is 30%. When the equivalence ratio *Φ* is constant, with the increase in the mixed argon content, and the average value of flame propagation speed decreases by 2.52–60.0%. The first and second deflagration pressure peaks are reduced by about 13.1–62% and 17.7–86.5% respectively.
3. The average velocity of flame propagation and the deflagration pressure can be significantly reduced by laying explosion-elimination chambers. The average flame propagation velocity is respectively reduced by 5.7%, 12.7%, and 37.0% under the condition of *Φ* = 0.9, *Φ* = 1.0, and *Φ* = 1.1, and the second and third pressure peaks caused by "external explosion" and "flame oscillations intensify turbulence" can be significantly reduced by 10–30% and 50–90% respectively.

## References

[1] Chen CC, Wang TC, Liaw HJ, Chen HC (2009). Nitrogen dilution effect on the flammability limits for hydrocarbons. J. Hazard Mater..

[2] Chen CC, Liaw HJ, Wang TC, Chen HC (2009). Carbon dioxide dilution effect on flammability limits for hydrocarbons. J. Hazard Mater..

[3] Degges MJ, Boyer JE, Kuo KK, Basinib L (2010). Influence of steam on the flammability limits of premixed natural gas/oxygen/steam mixtures. Chem. Eng. J..

[4] Kondo S, Takizawa K, Takahashi A, Tokuhashi K (2006). Extended Le Chatelier's formula for carbon dioxide dilution effect on flammability limits. J. Hazard Mater..

[5] Kondo S, Takizawa K, Takahashi A, Tokuhashi K (2006). Extended Le Chatelier's formula for nitrogen dilution effect on the flammability limits. Fire Saf. J..

[6] Bundy M, Hamins A, Lee KY (2003). Suppression limits of low strain rate non-premixed methane flames. Combust. Flame.

[7] Benedetto AD, Sarli VD, Salzano E, Russob G (2009). Explosion behavior of CH_4_/O_2_/N_2_/CO_2_ and H_2_/O_2_/N_2_/CO_2_ mixtures. Int. J. Hydrogen Energy.

[8] Mashuga CV, Crowl DA (1999). Flammability zone prediction using calculated adiabatic flame temperatures. Process. Saf. Prog..

[9] Shebeko YN, Fan W, BolodianI A, Navzenya VY (2002). An analytical evaluation of flammability limits of gaseous mixtures of combustible-oxidizer-diluent. Fire Saf. J..

[10] Vidal M, Wong W, Rogers WJ, Mannan MS (2006). Evaluation of lower flammability limits of fuel-air-diluent mixtures using calculated adiabatic flame temperatures. J. Hazard Mater..

[11] Cooper MG, Fairweather M, Tite JP (1986). On the mechanisms of pressure generation in vented explosions. Combust. Flame.

[12] Bauwens CR, Chaffee J, Dorofeev S (2010). Effect of ignition location, vent size, and obstacles on vented explosion overpressures in propane-air mixtures. Combust. Sci. Technol..

[13] Ponizy B, Leyer JC (1999). Flame dynamics in a vented vessel connected to a duct. Mechanism of vessel-duct interaction. Combust. Flame.

[14] Ferrara G, Benedetto AD, Salzano E, Russoa G (2006). CFD analysis of gas explosions vented through relief pipes. Hazard Mater..

[15] Solberg DM, Pappasj A, Skramstad E (1981). Observations of flame instabilities in large scale vented gas explosions. Sympos. Combust..

[16] Bradley D, Mitcheson A (1978). The venting of gaseous explosions in spherical vessels, I-Theory. Combust. Flame.

[17] Kasmani RM, Andrews GE, Phylaktou HN (2013). Experimental study on vented gas explosion in a cylindrical vessel with a vent duct. Process. Saf. Environ..

[18] Alexiou A, Andrews GE, Phylaktou H (1997). A comparison between end-vented and side-vented gas explosions in largeL/D vessels. Process. Saf. Environ..

[19] Wang Q, Guo ZR, Shen ZW (2012). Study on deflagration pressure distribution of combustible gas in PMMA square tube. Initiat. Pyrotech..

[20] Wang Q, Shen ZW, Guo ZR (2013). Analysis on propagation characteristics of premixed methane-air flame in an half-open tube based on high-speed video camera. Explos. Mater..

[21] Markstein GH (1964). Nonsteady Flame Propagation.

[22] Chen XX, Sun JH, Yao LY (2008). Characteristics of fine structure during tulip flame forming. J. Combust. Sci. Technol..

[23] Yu M, Yang X, Zheng K, Wan SJ (2018). Experimental study of premixed syngas/air flame propagation in a half-open duct. Fuel.

[24] Nian WM, Zhou KY, Xia CJ (2003). The experimental study of strength attenuation of gaseous etonation wave through acoustic absorbing walled channel. Fire Saf. Sci..

[25] Nie B (2011). The roles of foam ceramics in suppression of gas explosion overpressure and quenching of flame propagation. J. Hazard Mater..

